# Thohoyandou’s central business district and the hypothetical accessibility challenges for emergency services

**DOI:** 10.4102/jamba.v11i2.681

**Published:** 2019-06-24

**Authors:** Godfrey Anyumba

**Affiliations:** 1 Department of Urban and Regional Planning, School of Environmental Sciences, University of Venda, Thohoyandou, South Africa

**Keywords:** Central Business District, Urban Morphology, Emergency Services, Accessibility, Disasters, Mitigation

## Abstract

Thohoyandou is a town in the Limpopo province of South Africa, with a population of 69 453 according to 2011 Census. It has rapidly filled up the spatial limits of its central business district (CBD) to such an extent that the latest retail development, the Thavhani Mall, has leapfrogged the P-East residential area into the open spaces to the south of the CBD across the R 254. The objective of this study was to determine if emergency services - that is, police, ambulance and fire brigade - could access all parts of the CBD in a hypothetical situation of multiple disasters occurring simultaneously. The study method mapped the CBD’s urban morphological elements and determined, through qualitative descriptions, the frictional spaces each of the emergency services would face in attempting to access all parts of the CBD. The findings were, firstly, that in a ‘worst case scenario’ the emergency services would face formidable infrastructure, human and mobility obstacles in their pathways. The second finding is that the emergency services would not be able to cope with a high-impact disaster or a multiplicity of disasters. The study identified precautions that should be considered by the municipality and other stakeholders in order to mitigate the potential risks of human-induced disasters in the CBD.

## Introduction

Thohoyandou is a small town in the northern part of Limpopo province in South Africa, with a population of 69 453 people as per 2011 Census. This article is about a localised scale of potential man-made disasters in the town’s central business district (CBD). The article takes on the concept of disaster risk management from the perspective of urban design. It seeks to illustrate how an evolved urban fabric may lead to a gradual closing of accessibility options for emergency services when a disaster risk is looming. The article argues that urban morphology and patterns of human activities will impact the ability of civilian emergency services to function as intended. The value of this type of disaster management study lies in its propensity to point out, at the conceptual level, the basis of hazard mitigation plans.

In a study on the risk and vulnerability of South African settlements, Van Huyssteen, Le Roux and Van Niekerk (2013) pointed out the need for an integrated ‘place-specific’ approach to disaster risk and management. This vulnerability awareness building process may contribute to local authorities adopting more robust town planning schemes and urban design frameworks. The results of the present study specifically inform accessibility pathway challenges for emergency services.

Within the disaster risk and disaster management discourse, the former United Nations Secretary General, Kofi Annan (2002), observed amongst others that:

Communities will always face natural hazards, but today’s disasters are often generated by, or at least exacerbated by human activities […]. Poor land-use planning; environmental management; and a lack of regulatory mechanisms both increase the risk and exacerbate the effects of disasters. (p. 1)

From these observations this article construes its rationale for an urban-focused disaster risk and disaster management theme.

For the purposes of contextualising its aims, this article adopts the United Nations International Strategy for Disaster Reduction (UNISDR) definition of vulnerability, which is (Annan 2002):

[*T*]he potential for loss (human, physical, economic, natural, or social) due to a hazardous event. It is the characteristics and circumstances of a community, system or asset that make it susceptible to the damaging effects of a hazard. (n.p.)

A framework for disaster risk management was developed at the 2005 World Conference on Disaster Reduction. It is stated that the framework was developed in the Hyogo Framework for Action (2005–2015): *Building the Resilience of Nations and Communities to Disasters*. It was summarised by a five-point ‘to do’ list (see Table 1).

**TABLE 1:** Hyogo Framework for Action disaster mitigation themes.

Building the resilience of nations and communities to disasters
1. Ensure that disaster risk reduction is a national and a local priority with a strong institutional basis for implementation
2. Identify, assess and monitor disaster risks and early warning
3. Use knowledge, innovation and education to build a culture of safety and resilience at all levels
4. Reduce the underlying risk factors
5. Strengthen disaster preparedness for effective response at all levels

*Source*: UNISDR 2009

The central theme of this article adopted part of an extract from the second of the five Hyogo Framework for Action themes, which states that (Hyogo Framework for Action 2005–2015):

The starting point for reducing disaster risk and for promoting a culture of disaster resilience lies in the knowledge of the hazards and the physical, social, economic and environmental vulnerabilities to disasters that most societies face, and of the ways in which hazards and vulnerabilities are changing in the short and long term, followed by action taken on the basis of that knowledge. (p. 7)

This study is limited to the case of identifying and accessing disaster risks at Thohoyandou’s CBD.

The article adopts the viewpoint that a man-made disaster would be constituted if an ‘out of control’ human-induced situation was to occur with the consequent loss of life and the destruction of property. However, there are no records of disasters, such as riots or multiple robberies of banks or malls, in the history of Thohoyandou’s CBD. However, there have been several serious fires that destroyed Shoprite supermarket, Madina supermarket and Nandos since the establishment of the CBD in 1977 (Dzebu 2006; Tshikhudo 2008).

This article argues that police, ambulance and fire brigade services need to access a settlement’s built fabric timely to execute their emergency functions. If the services’ ability to penetrate the built form is compromised for any reasons, then the situation of disaster risk is greater. This is the rationale behind undertaking this investigation.

The remainder of this article is organised as follows: firstly, the genesis of Thohoyandou’s CBD is presented, followed by the problem statement, the purpose of the study, the methodology used and an urban morphological analysis. A hypothetical ‘worst case scenario’ emergency is built in as the method of determining the risks that emergency services would face. The obstacles to the three emergency services are individually determined. The study rounds off the investigations by presenting a cluster of emergency services accessibility challenges, disaster mitigation measures and the conclusions.

## Genesis of Thohoyandou’s central business district

**FIGURE 1:**
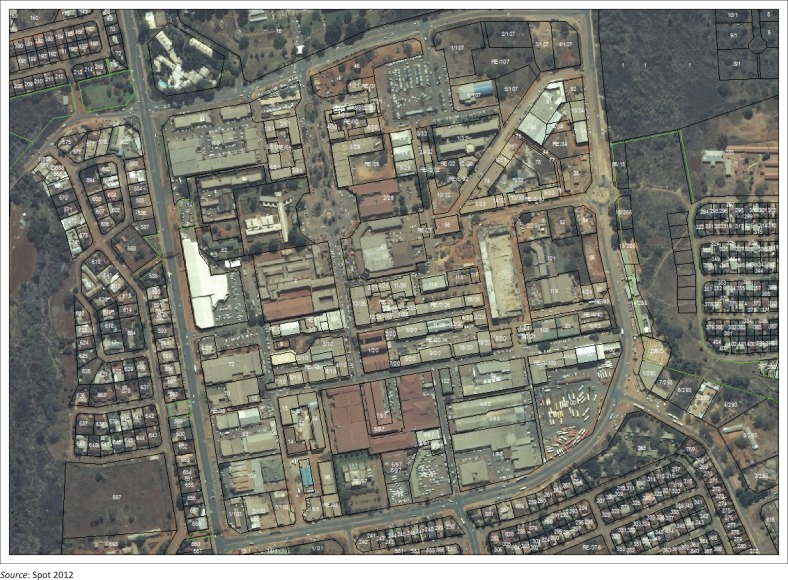
Thohoyandou central business district 2012.

The study location, CBD, is a relatively small but intensively built up area. It covers an area of approximately 70 ha (see Figure 1).

The structure of Thohoyandou’s CBD is inextricably linked to the urban history of the town. According to VhaVenda History (n.d.), Thohoyandou town was established on a portion of the Mbaleni village. The more or less grid structure of the CBD had its beginnings in 1977. The initial government buildings were erected with the establishment of the Republic of Venda in 1979, to the north of the present CBD. For many years the CBD contained other government services, such as the post office, police station, courts, as well as wholesale and retail outlets, taxi ranks and bus stations.

During the Venda Republic era, the CBD and the neighbouring residential areas were demarcated through the work of South African consultants supported by South African Government finances. Dr Nethengwe (pers. comm. 23 February 2015), a long-time resident of Thohoyandou, confirmed the above facts. However, what has been consistent in the CBD’s physical demarcation is the ring road that separates the CBD from the government buildings, the forest to its north, the wetland to the east and the residential-come-business areas to the west (P-West) and south (P-East) of the ring road.

Today the CBD is dominated by single- and two-level buildings, with a few vacant erven (plots). There is very little in terms of green areas in the CBD. Presently, the growth of central Thohoyandou is unabated, with new building construction evident outside of the CBD ring road. It can be surmised that the growth of the CBD has been undoubtedly positive for the banking, retail and wholesale sectors, as well as for the informal street traders.

**FIGURE 2:**
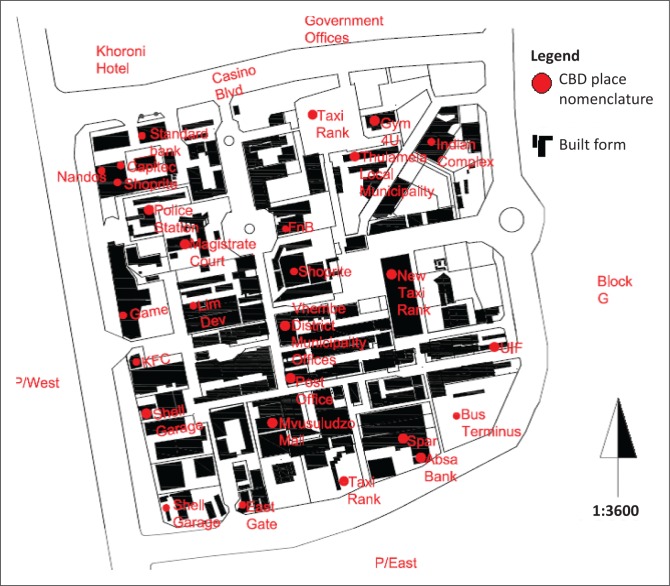
Central business district place nomenclature. CBD, central business district.

Like many former R 239 towns, Thohoyandou has no street names to speak of. The absence of precise geographical place and street nomenclature presents problems for emergency services, not to mention the ordinary citizen. Figure 2 presents the CBD place nomenclature.

## Problem statement

The problem statement of this study is based on a ‘worst case scenario’, where a multiplicity of human-induced disasters occur concurrently. The question is as follows: would the emergency services gain the requisite level of accessibility into Thohoyandou’s CBD? Thus, the problem statement is hypothetical.

### Purpose of the study

The purpose of this study was to ascertain the extent to which the current spatial footprint would act as impediments to the access of any parts of the CBD for emergency services. In other words: does the situation enhance or diminish the effectiveness of emergency services in executing their functions in disaster risk reduction?

### Methodology

The study methodology consisted of several approaches. Firstly, an urban morphology approach with specific reference to figure-ground spatial mapping was undertaken for (1) the underlying erven structure, (2) the built form, (3) open spaces and parking spaces, (4) street-level obstacles and (5) street trading areas. The purpose of the application of this approach was to determine how the properties inherent in the above morphological schemata may impact emergency vehicular and pedestrian accessibility to all parts of the CBD.

Secondly, a ‘worst case scenario’ under which the three emergency services would find themselves was constructed to test the ability of the services to deliver under stressful emergency conditions.

Thirdly, all service providers, that is, police, ambulance and fire brigade, were examined in terms of their accessibility within the CBD. Notes were taken of any particular characteristics that hindered their full coverage of the CBD.

Lastly, a matrix was constructed highlighting the hazards generated in a worst case scenario. Open-ended interviews with some of the service providers were also undertaken.

### Urban morphological analysis

A figure-ground approach to the Thohoyandou CBD was constructed from a 2012 Thohoyandou BA Locality Map. Urban morphology construction depicted underlying properties of each element of urban form as they apply in the CBD.

#### The underlying erven structure

**FIGURE 3:**
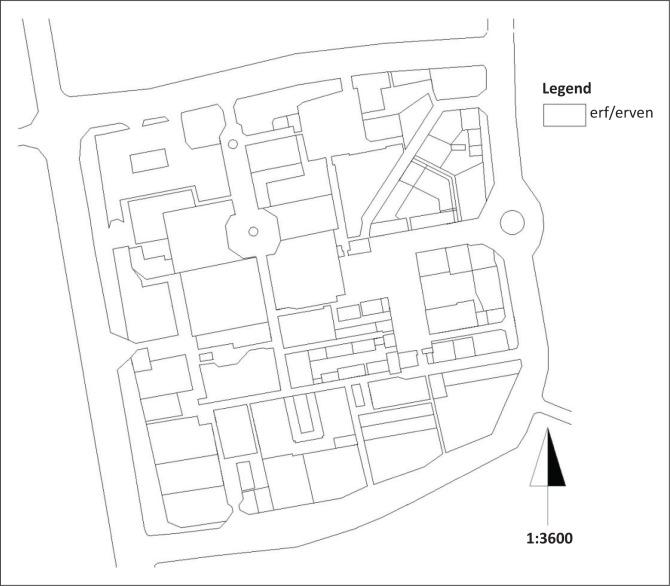
Underlying erven structure.

Except for the north-east of the CBD where the erven or plots are triangular, the rest are rectangular and square in shape. There are three basic sizes that can be categorised as (1) small-sized erven (150 m^2^–1800 m^2^), (2) medium-sized erven (2100 m^2^–8500 m^2^) and (3) large-sized erven (9000 m^2^–19 000 m^2^) (see Figure 3).

The rule of thumb is that the smaller the erven, the smaller the corresponding footprint of buildings that can be erected on them and the greater the accessibility of services, all other factors being equal, from its street frontage and backyard, etc. The larger size erven are ideal for the erection of large buildings, such as shopping malls, police stations and municipality buildings, or for their open space designation as ceremonial or public squares.

The majority of Thohoyandou’s erven sizes are large and the implications are that the majority of buildings are of a large footprint. Geo Ville defines building footprint as ‘the aerial visible first floor projection at grade to the edge of built area including conditioned and non-conditions spaces: living area, decks, garages, porches, etc.’ (Geo Ville n.d.:n.p.).

#### The built form

**FIGURE 4:**
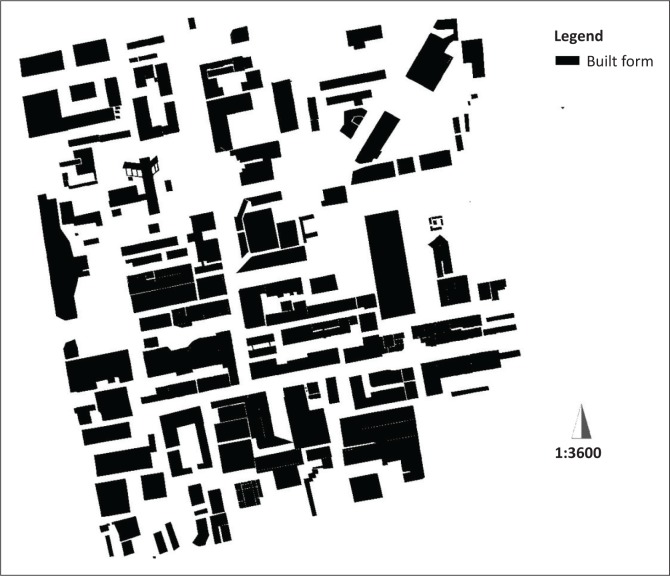
The built form of buildings in the central business district.

The built form is the shape of a building (concrete footprint) as it sits astride its erf/plot. The built form of Thohoyandou’s CBD illustrates several features. The majority of buildings tend to have a comparatively large footprint. Buildings on the whole are ‘stand-alone’ or ‘detached’ in configuration. Buildings have considerable length and shallow depth. Most of the buildings are configured with two or three interfaces (frontages) with streets (see Figure 4).

The impacts of the built forms and urban fabric for ‘emergency services penetration’ vary according to the sizes of buildings and their layout. For example, larger buildings present potential challenges for the firefighting dousers to reach their mid or upper levels, while buildings with smaller footprints are better predisposed to coverage by emergency services.

#### Open spaces and parking spaces

**FIGURE 5:**
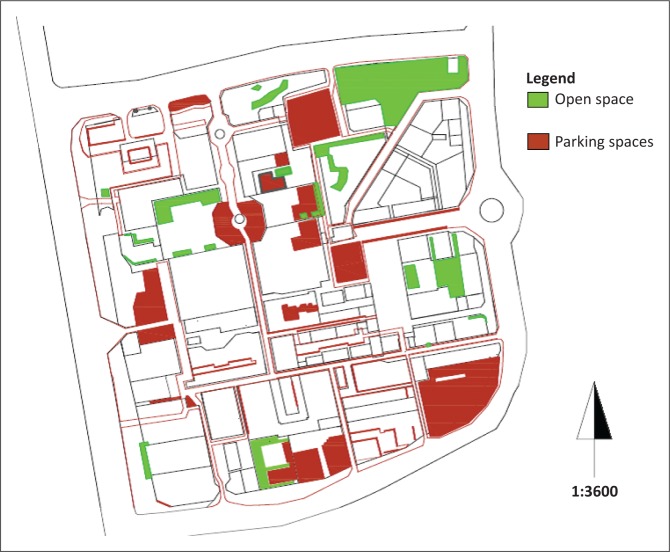
Open spaces and parking spaces.

There is very little left of green open spaces in Thohoyandou’s CBD; however, open and parking spaces can play both positive and negative roles in a disaster situation. Either space can be utilised to set up temporary placements, for example, landing spot for an aerial evacuation, a detention centre for riot control or a space for a tented clinic, etc. However, open space vacancy may attract unwarranted crowds during a disaster. Their spatial distribution is shown in Figure 5.

Car parking spaces that run parallel to streets normally tend to be fully occupied with vehicles by mid-morning. However, there are also illegal parking ‘extensions’ by desperate vehicles seeking space. A second type of parking space represents those clustered in areas adjacent to large shopping malls. Examples include car parking space next to Game or Shoprite at Venda Plaza. With their normally high occupancy rates, parking spaces would present obstacles to emergency services. Taxi ranks and the bus terminal are characterised by recurring overspill onto the ring road that disrupts normal traffic circulation. These traffic movements would definitely impede the movement of emergency services into the interior of the CBD.

The verges of car parks in the CBD are occupied by street traders, who are strategic in their choice of locations (Anyumba 2001; Tapela 1999). The practice of occupying these spaces is prevailed throughout the CBD. Street traders and their portable street furniture would be obstacles to the movement of people and vehicles during emergencies.

#### Street-level obstacles

**FIGURE 6:**
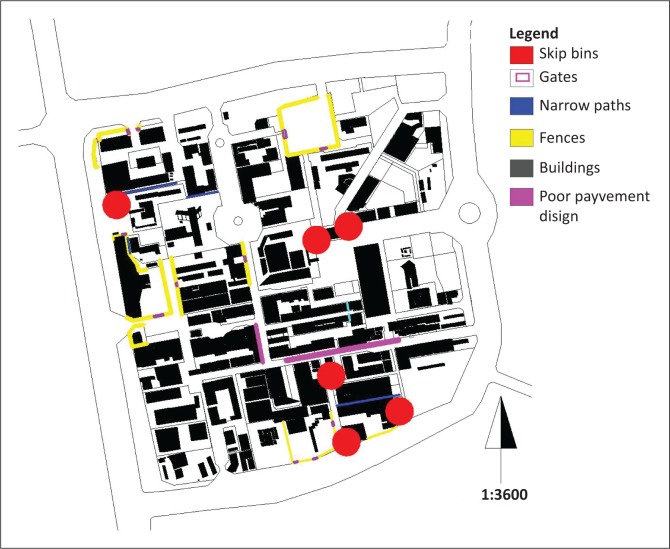
Street obstacles in the central business district.

In the CBD, there are physical immobile street-level obstacles that would be a nuisance to the movement of emergency service providers’ equipment and vehicles. Potential obstacles include (1) cul-de-sac and one-way streets, (2) fences and gates and (3) poor placement of street furniture (see Figure 6).

In the CBD, there is a single ‘cul-de-sac’ south of the police station and a one-way street to its north. The consequences of cul-de-sac and the one-way street are that emergency services must in advance be aware of where their locations are and maybe make use of them in an emergency.

Possibly the most common permanent obstacles are the fences (1.5 m high) and gates within the west of the CBD. This was not a feature of the town centre until recently. In an emergency, these features could prove to be formidable obstacles for the movement of equipment and emergency personnel.

Poor street furniture such as street lamps, narrow streets, manhole covers higher than the street level and steep stepped pavements are obstacles that have rendered parts of the CBD inaccessible to certain sizes of equipment and people.

A number of overflowing skip bins are a fire hazard because of the exposed combustible wastes contained therein. Another fire hazard is the timber placed in front of hardware stores that are also obstacles to pedestrian movement along pavements.

#### Street trading

**FIGURE 7:**
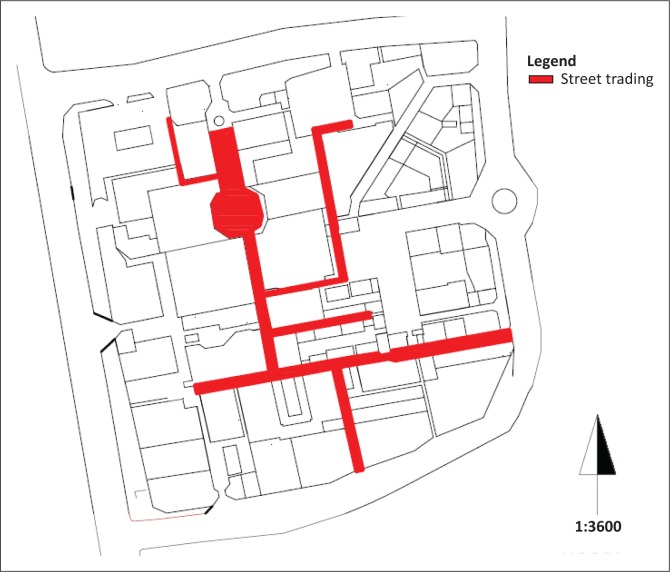
Street trading areas.

Despite their recognised contribution to South Africa’s economy (Smith 2014), under the present circumstances street trading organisations in the CBD that are licensed to occupy certain spaces would nevertheless be an obstacle to emergency services. Figure 7 illustrates the extent of street trading activities that occupy the above referred spaces.

### Assessing Thohoyandou central business district’s accessibility

Litman (2015) highlights the fact that accessibility is interpreted in different ways in different disciplines. The study’s adopted definition is the meaning ascribed by ‘geography and urban economics’… where, ‘*accessibility* refers to the relative ease of reaching a particular location or area’ (Litman 2019). Litman notes that land use planners generally focus on geographic accessibility, that is, distances between activities.

This article asserts that no part of Thohoyandou’s CBD should be beyond the accessibility requirements of emergency services. If any service cannot access a certain area where it needs to render its services, then it is failing and thus may not be able to respond to the disaster it is called upon to control and eliminate. For the purposes of this study, the accessibility requirements are defined under each of the emergency services in the following sections.

### Worst case disaster scenario

This article assumes the conditions of worst case scenario, which include multiple disasters during a hypothetical festive season when the CBD is characterised by the following conditions occurring simultaneously over a number of daytime hours:

Legal and illegal car parks occupy 99% of spaces, the taxi ranks and the bus terminus are in grid lock, and festive people occupy 90% of left-over pedestrian spaces. Furthermore, there are a great number of physically challenged people in every street in a celebratory mood, and street traders are occupying all the open spaces they can access for maximum trading opportunities. Meanwhile, robberies by well-armed and violent gangs are going on in two of the largest shopping malls and there were five substantial fires started by arsonists in street bins and shop fronts with flammable materials. Last but not least, some security commander has ordered the closing of all gates to contain the situation.

In the following sections, the study points out the extent to which the three emergency services would be able to reach any point in the CBD.

#### The South African Police Service

The South Africa Police Services (SAPS) station is strategically located in the centre-west of the CBD. Besides the assumption that police officers should be able to penetrate and secure any building or any open space, other expectations include the following: (1) that police officers would be either on foot patrol or driving police vehicles and (2) that the SAPS would be able to communicate effectively between themselves and can decipher any hostile communications in the environment (Anyumba 2003).

**FIGURE 8:**
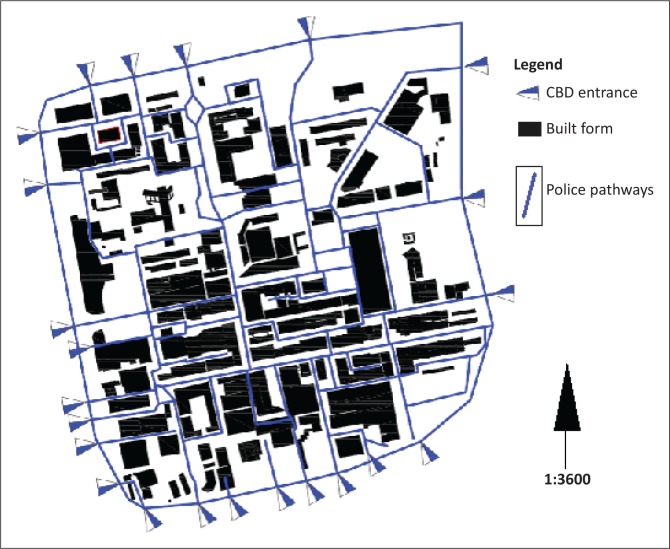
Police pathways. CBD, central business district.

In evaluating the penetration of the physical structure of the CBD, the study investigated pathways for police foot patrols and police vehicles. Further assumptions were that (1) the police would depart from the police station and (2) the police could approach any part of the CBD from any vantage point using the CBD ring road. The police pathways (Figure 8) shows that foot patrols and vehicles would be in a position to penetrate any part of the CBD. There are 23 drivable entries into the CBD: four from the north, three from the east, eight from the south and eight from the west. The number of foot patrol entrance points into the CBD is beyond counting.

Nevertheless, a number of physical and human obstacles, such as fences, closed gates, narrow lanes, cul-de-sac, crowds of people, mobile and parked motor vehicles, taxi ranks and buses and buildings, could conceal dangers as the SAPS criss-crosses the CBD (see Table 2).

**TABLE 2:** Hazards generated from ‘worst case scenarios’ for the police service.

Emergency services	Emergency locality situation					
	Obstacles		Circulation			Hazardous materials/ conditions
	Vertical	Horizontal	Visual-2D views	Vehicle	Pedestrians	
Urban morphological elements	Fences (1.5 m), walls and open spaces	Steps and road and building surface intrusions	Obtrusive buildings, structures and mature trees Cul-de-sac	Uncontrolled vehicular traffic and legal and illegal parking	High human shopping and street trader densities	Potholes, abandoned kiosks and goods
Foot and vehicle patrol	Climbing and descending, steep steps and crawling over walls	Potholes slow traffic	Street furniture and informal traders’ structures	Traffic jam Parking spaces fully occupied	Human spatial friction and the curiosity factor	Concealed traps

2D, two-dimensional.

#### Ambulance services

Complicating the role of ambulance services and the CBD situation is that there is no hospital or a large medical facility within Thohoyandou’s CBD. Therefore, any ambulance attending to the needs of the CBD population would have to drive from (1) the University of Venda Clinic (5–7 min drive away), (2) Tshilidzini Hospital (8–10 min drive away) and (3) Donald Frazer Hospital (30–45 min drive away) or Elim Hospital (at least 1 h drive away).

**FIGURE 9:**
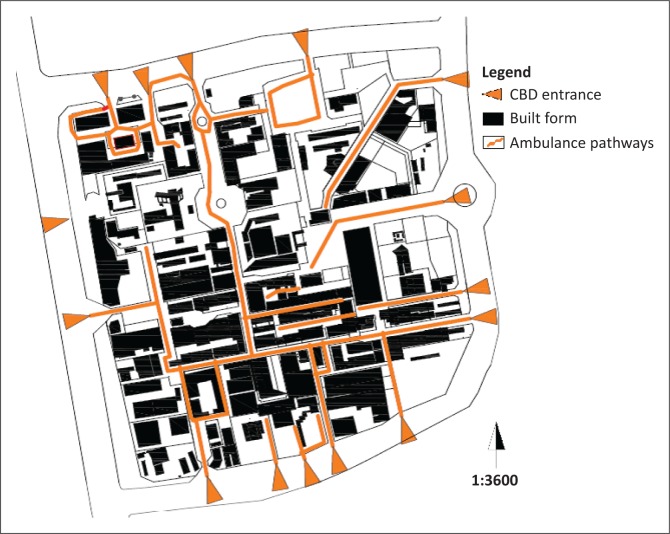
Ambulance pathways. CBD, central business district.

Figure 9 illustrates that there are a total of 15 possible entry points from the ring road for ambulances. There is one entrance in the northern ring road that accesses the built-up area past its geographical centre and one from the east that almost crosses to the west of the CBD.

The CBD shows that ambulances would face some serious obstacles arising from the configuration of the built form. Examples include the north-west corner of the CBD where an ambulance would have to drive in circles to reach its destination. To the south and south west, the continuously built-up area acts as another set of barriers. A large part of the CBD would require ambulance personnel to move with stretchers and medical equipment on foot. Other problem areas are summarised in Table 3.

**TABLE 3:** Hazards generated from ‘worst case scenarios’ for ambulances.

Emergency Services	Emergency site situation					
	Obstacles		Circulation			Hazardous conditions/materials
	Vertical	Horizontal	Visual-2D views	Vehicle	Pedestrian	
Urban morphological elements	Fences (1.4 m), walls and open spaces, Bollards	Steep steps, building and surface/path intrusions	Obtrusive buildings, streetscapes and landscapes	Lack of street names	High human shopping and street trader densities	Intoxicating fumes
Vehicles	-	Potholes slow emergency traffic, may cause injuries to evacuees	Unable to determine situation in the third dimension	Vehicle passages, spaces for parking and orientation issues	Accident curiosity factors slow vehicle movement	
Portable equipment	Equipment may be damaged or too heavy to carry by non-motorised means	Wade through steep stepped streets	Emergency crews unfamiliar with layout may be lost in the streets	Street furniture and informal traders’ structures slow emergency vehicles and personnel	‘Help’ from the public may obtrude mission	There may be insufficient equipment to deal with the numbers of people requiring attention

2D, two-dimensional.

#### Fire brigade

Vhembe District Municipality is responsible for this service. Thohoyandou’s CBD has had several significant fires in the recent past that destroyed several retail and food outlets. These include the Shoprite building that was burnt down in 2006, Nandos restaurant in 2008 (Tshikhudo 2008) and a hardware store south of Game in 2013. It is claimed that the causes of all these fires were related to electrical faults.

In 2015, the fire brigade had three functional fire engines, two of which were less than five years old and one was much older than the other two. When interviewed on 08 February 2017, Mr T.M. Makumule, a former Vhembe District municipal manager, stated that the fire engines did not require ladders as the tallest building in the region was only three floors high. It was noted that the fire engines had an internal water capacity of 8000 L each.

The CBD has three fire hydrants located at (1) the old Shoprite shopping complex at the centre of the CBD, (2) the UIF Building to the east of the CBD and (3) Game to the west of the CBD. Because the fire station is located to the southwest of the CBD, the other assumptions are that (1) fire brigade vehicles would approach the CBD from the R524, (2) that vehicles would be able to reach each fire hydrant and (3) vehicles would have sufficient spread to cover any CBD building or open space.

**FIGURE 10:**
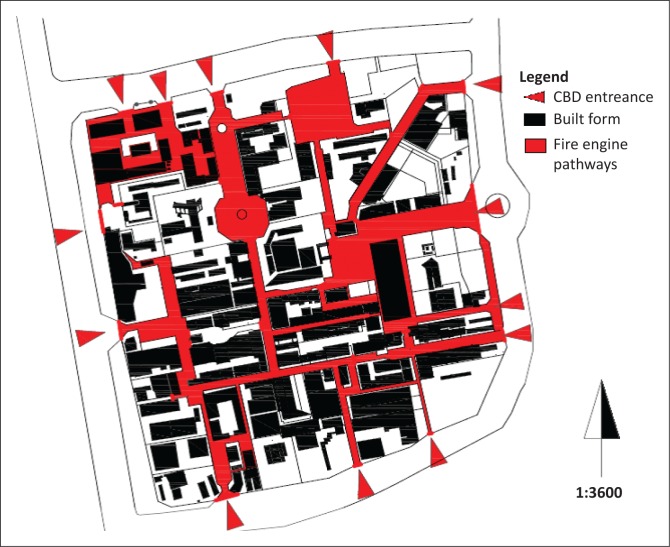
Fire engine pathways. CBD, central business district.

From the above cited interview of Mr Makumule, it is clear that fire engines are able to negotiate all the streets within the CBD (see Figure 10). The major obstacles that the fire engines would face when dowsing out fires would be crowd control and having to navigate through parked and moving vehicles. Mr Makumule also noted a number of challenges. Firstly, the current water reticulation system does not function for 24 h a day because the water supply to the CBD is open during the day and gets turned off at night when it is empty of people, so that residential areas outside of the CBD can be supplied with water. Secondly, Nandoni dam, which started operations in 2005 and has a water capacity of 164 million cubic meters, is still to be connected to provide a more permanent supply of water to the CBD and other parts of Thohoyandou. Each building is required by regulation to have fire extinguishing equipment. A rapid visual assessment of the buildings in the area revealed that the required firefighting equipment was generally not in place. However, some fire extinguishers were clearly visible in a number of newer buildings in the CBD. Other problem areas are outlined in Table 4.

**TABLE 4:** Hazards generated from ‘worst case scenarios’ for the fire brigade.

Emergency services	Emergency site situation					
	Obstacles			Circulation		Hazardous conditions/materials
	Vertical	Horizontal	Visual-2D views	Vehicle	Pedestrian	
Morphological elements	Fences (1.4 m), walls and open spaces, bollards, tall buildings (three level)	Lengthy or bulky buildings	Unable to determine situation in the third dimension	Lack of street names Parked vehicles	High human shopping and street trader densities	Solid waste materials on pavements
Vehicles	-	Potholes slow emergency traffic and may cause injuries to patients	Smoke concealment Obtrusive buildings	Ability to turn into confined sites	People curious about fires may slow fire engine movement and endanger themselves	Combustible materials in streets
Portable equipment	Street/ pavement levels	Street barriers – fencing	Smoke concealment	-	Unhelpful curiosity factor	The lack of equipment to deal with hazards
Site Equipment	Water hydrants malfunction	-	-	-	-	-

2D, two-dimensional.

### Accessibility challenges in the central business district

From the urban morphological analysis, field observations and interviews, it became apparent that the three emergency services would struggle to successfully meet the ‘worst case scenario’ accessibility requirements outlined above.

Accessibility challenges common to all the three services include physical barriers, densities of parked vehicles, traffic jam and the human curiosity factor (on part of shopkeepers, street traders and the general public). These human-induced barriers could definitely result in unwarranted interference with emergency service providers’ work and pace of movement.

Whilst the fire brigade shares the common constraints noted above, the brigade as presently constituted would encounter the following problems. The first is that if fires were to break out in those parts of the CBD that have no fire hydrants (and there are only three in the entire CBD), this would present immense firefighting difficulties. Further complications would arise if the fire hydrants are dry! Still the fire engines would have 2000 L – 6800 L water in their tanks for the emergency; however, if this source of water is exhausted, then such a vehicle would need to refill its tank – a process that has to take place outside of the CBD. If there is no water in the CBD and in the adjacent neighbourhoods, then the last recourse would be to drive to Nandoni dam, a distance of 18 km from the CBD. Last but not least, if there are more than three fire incidents at the same time, then the full capacity of the fire brigade would be exhausted and help would be required from Louis Trichardt in Makhado Municipality, a distance of 70 km and at least 1 h drive.

### Disaster mitigation measures

In the above sections, we have identified and assessed some of the disaster risks that the three emergency services would face in a worst case scenario in Thohoyandou’s CBD. Mitigation measures are actions that aim to completely erase or reduce the severity of disasters.

The following disaster mitigation measures are suggestions that are informed by the urban morphology and human situational analysis expounded above, which the district and local municipalities, the emergency services and all stakeholders should take cognisance of, including building plan approval, street design, vehicle control, emergency equipment, public warning systems, crowd control (practicing of emergency drills and open spaces).

#### Building plan approval

When building plan approval is undertaken, in the CBD and elsewhere, emphasis should be given to the fact that the challenges identified above, that is, unhindered accessibility of emergency services, are addressed. Thus, building approval must consider the building footprints with adjacent existing or planned buildings and with open spaces. In other words, plan approval should not only consider the architectural and the land use plans but also the total conceivable urban design of a planned situation.

#### Street design

Certain streets in the CBD require interventions to ensure increased safety of pedestrians, especially physically handicapped persons. A section of the CBD that sells timber and hardware goods should perhaps be relocated, where offloading bay for articulated trucks can be accommodated. Otherwise, these large trucks tend to block streets when parked or when offloading freight.

#### Vehicle control

Visual circumspection shows that the CBD has definitely run out of public parking space, with peak parking time extending from 10:00 to 17:00. Parking and movement within these spaces are a challenge, with vehicles spilling out into the local and distributor roads in the CBD. Private vehicles, taxis and buses tend to block traffic. The Thulamela Traffic Police must find a means to control the present situation. Developers should consider feasibility studies for building multilevel car parks in the CBD.

#### Emergency equipment

As part of disaster mitigation, the three emergency service providers should define equipment that would assist in executing their functions, which need to be in place in the CBD. These may include public address systems and surveillance systems linked to the police services, ambulances and the fire brigade. Other equipment, as required by law, should be enforced. These may include fire extinguishers, more water hydrants and first-aid kits, etc. Such equipment should be strategically located in public places within the CBD.

#### Public warning systems

There should be alarm systems that could alert the public on impending disaster or a state of emergency. Such a system should be located in strategic public places.

#### Crowd control: Practice of emergency drills

In building public awareness, the relevant authorities should undertake the practice of emergency evacuation drills from time to time. By undertaking such exercises, the population in the CBD can be educated to conduct themselves in the correct and safe manner.

#### Open spaces

Open spaces, both green and hard surface, should be designated around clusters of buildings and/or shopping areas that may become assembly points in the case of an emergency or a disaster.

## Conclusions

Thohoyandou’s CBD has not experienced any civil unrest that has threatened life and property in its 40 years history. However, it has experienced several serious fires between 2006 and 2009. An urban morphological analysis of the configuration and infrastructure in the CBD, as well as observations of the characteristics of its street trade and vehicular parking, shows that the three civilian emergency services (i.e. police, ambulance and fire brigade services) would face a range of obstacles that would slow the performance of these services if a serious disaster was to break out. Under a theoretical ‘worst case scenario’ of a multiplicity of disasters occurring simultaneously, it was illustrated that the emergency services would have a difficult time gaining access to the disaster areas. The study findings point to the need of a certain line of disaster mitigating policies, appropriate infrastructure and sensitising of the daytime occupants of the CBD in preparation for managing any disaster. The limitations of the study included the lack of expertise and time to quantify the frictional basis that would inhibit the deployment of emergency services successfully.
